# Selection in Coastal *Synechococcus* (Cyanobacteria) Populations Evaluated from Environmental Metagenomes

**DOI:** 10.1371/journal.pone.0024249

**Published:** 2011-09-09

**Authors:** Vera Tai, Art F. Y. Poon, Ian T. Paulsen, Brian Palenik

**Affiliations:** 1 Scripps Institution of Oceanography, University of California San Diego, La Jolla, California, United States of America; 2 British Columbia Centre for Excellence in HIV/AIDS, Vancouver, British Columbia, Canada; 3 Department of Chemistry and Biomolecular Sciences, Macquarie University, Sydney, New South Wales, Australia; Universidad Miguel Hernandez, Spain

## Abstract

Environmental metagenomics provides snippets of genomic sequences from all organisms in an environmental sample and are an unprecedented resource of information for investigating microbial population genetics. Current analytical methods, however, are poorly equipped to handle metagenomic data, particularly of short, unlinked sequences. A custom analytical pipeline was developed to calculate dN/dS ratios, a common metric to evaluate the role of selection in the evolution of a gene, from environmental metagenomes sequenced using 454 technology of flow-sorted populations of marine *Synechococcus*, the dominant cyanobacteria in coastal environments. The large majority of genes (98%) have evolved under purifying selection (dN/dS<1). The metagenome sequence coverage of the reference genomes was not uniform and genes that were highly represented in the environment (i.e. high read coverage) tended to be more evolutionarily conserved. Of the genes that may have evolved under positive selection (dN/dS>1), 77 out of 83 (93%) were hypothetical. Notable among annotated genes, ribosomal protein L35 appears to be under positive selection in one *Synechococcus* population. Other annotated genes, in particular a possible porin, a large-conductance mechanosensitive channel, an ATP binding component of an ABC transporter, and a homologue of a pilus retraction protein had regions of the gene with elevated dN/dS. With the increasing use of next-generation sequencing in metagenomic investigations of microbial diversity and ecology, analytical methods need to accommodate the peculiarities of these data streams. By developing a means to analyze population diversity data from these environmental metagenomes, we have provided the first insight into the role of selection in the evolution of *Synechococcus*, a globally significant primary producer.

## Introduction

Circumventing the need for cultured isolates, environmental metagenomics provides genetic sequences from microorganisms of entire communities. The collections of sequences reveal simultaneously the breadth of gene content and genetic diversity of these communities. Environmental metagenomes have been obtained from a wide diversity of ecosystems, all driven by interest in the biogeochemistry, taxonomic diversity, evolutionary history, and ecology of the microbial community [1]–[6].

The sampling and analyses of environmental metagenomes can be customized to examine taxonomic groupings at any phylogenetic or ecological scale. This is an unprecedented resource, especially for microbial population biology. As each metagenome sequence is derived from an individual microbe, the diversity of native microbial populations exists within the metagenome datasets. Population analyses of microbes were previously limited to sequences of marker genes or genomes of cultured isolates (see for eg. [7], [8]). Environmental metagenomes contain a census of the true population diversity, genome-wide, without bias with respect to culturability.

The population is the fundamental unit upon which selection acts. Individuals in a population have varying phenotypes, but those with fittest phenotypes have a greater chance of survival and reproduction. Variations in genetic sequences, acquired through mutation or gene transfer, provide the raw material for phenotypic variants of a given trait. While mutation increases variation, selection and genetic drift act to reduce it.

The field of population genetics provides the theoretical framework to examine the effects of mutation, selection, and drift on the diversity, evolution, and demographic history of a population. One method of investigating the role of selection in the evolution of a gene in a population is to determine the dN/dS ratio [9]–[12]. This ratio tests whether the nucleotide variants (polymorphisms) of a gene in a population contribute to an excess of non-synonymous mutations (mutations that change the amino acid sequence) than expected by chance (dN/dS>1). This would imply that the gene is undergoing positive selection as natural selection is favoring more diversification at the amino acid level. Purifying selection is characterized by fewer amino acid changes than expected by chance (dN/dS<1). Most genes are thought to have evolved this way as most mutations are deleterious and removed by selection, and the occurrence and fixation of beneficial mutations is rare [13]–[16].

Only a handful of studies have used environmental metagenomic data to investigate the population genetics of microbial communities [17]–[20]. These investigations all demonstrate the predominance of purifying selection in the evolution of microbial populations. By analyzing Sanger-based sequences, these previous studies could take advantage of methods already established for population genetics analysis. Several approaches to calculate dN/dS rely on pairwise alignments or multiple sequence alignments to first infer a phylogenetic tree from which the evolutionary path of mutational changes is assessed [21]–[25]. These methods, however, are ill equipped to handle the short sequence reads (<500 bp) of next-generation sequencing technologies, such as Roche 454 or Illumina. With short sequence reads, especially if linkage data is unavailable such as from environmental metagenomes, obtaining lengthy sequence alignment blocks (all sequences aligned to the same region) across a gene is not possible.

This article examines the population diversity of *Synechococcus* from coastal samples of the Southern California Bight – an environment dominated by *Synechococcus* from two clades, I and IV [26]. The role of selection in the evolution and diversification of these *Synechococcus* populations was examined by developing new analytical methodologies to calculate dN/dS ratios from metagenomes of Roche 454 sequences. The dN/dS ratios calculated here were used simply to characterize the diversity observed from the *Synechococcus* populations as opposed to more sophisticated methods which require phylogeny, linkage data, and population boundaries to be known. Marine *Synechococcus* are a cyanobacterial group with major roles in oceanic primary productivity and the global carbon cycle [27]–[30]. The methods we report here were applied to *Synechococcus* populations but are of general use for examining microbial population genetics.

## Results

**Figure 1:**
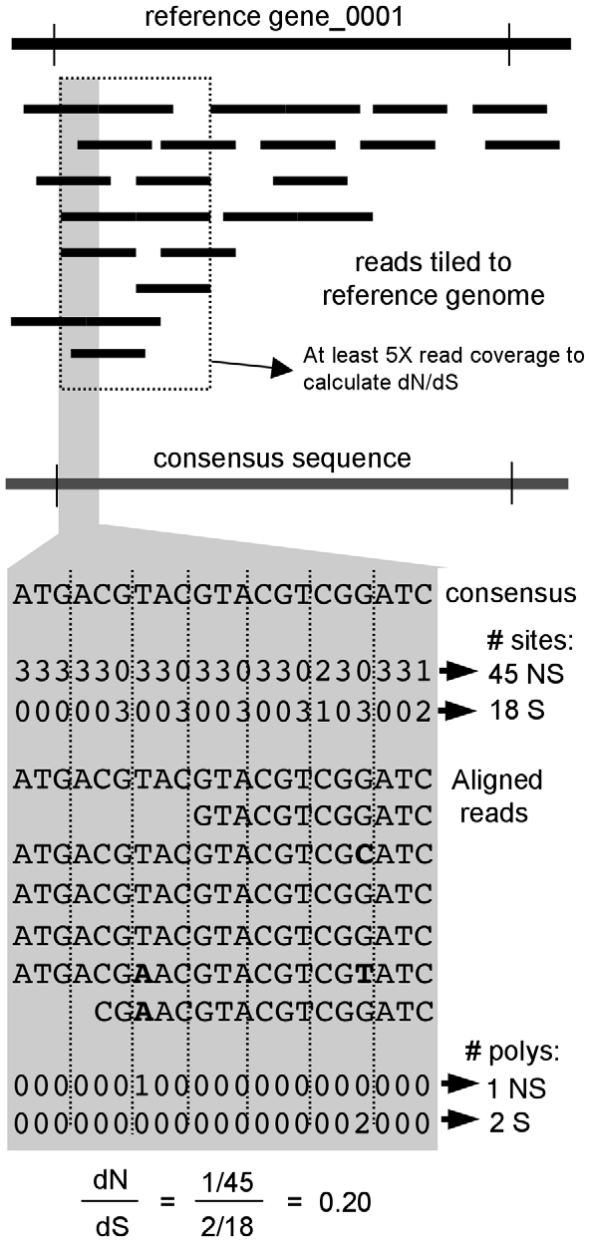
Schematic of environmental metagenome tiling and dN/dS calculation. Sequence reads were tiled to a reference genome. A majority-rule consensus sequence was generated from the aligned reads. The number of non-synonymous (NS) and synonymous (S) sites were calculated from the consensus sequence. The observed NS and S polymorphisms (polys) in the aligned reads relative to the consensus sequence are shown in bold. An example of aligned reads and dN/dS calculation is shown for a small section of a gene, but the same methodology would be used for an entire gene.

The approach for calculating dN/dS ratios using environmental metagenomic 454 reads was to identify single nucleotide polymorphisms in the environmental 454 reads relative to an environmental consensus sequence (Figure 1). The 454 reads were aligned to a reference genome to identify the reads belonging to a “population”. A threshold of genetic similarity with the reference genome defined this “population”.

Depending on the platform used, the 454 sequences analyzed here averaged 100 or 250 bp in length. Thus, individual sequence reads could not cover the length of a gene and the aligned sequence reads were staggered across the gene in varying depths of coverage. Furthermore, the 454 reads could not be linked genetically. Therefore, pairwise approaches to calculating dN/dS ratios could not be used practically as the sequences are required to align to the same genetic region, *i.e.* an alignment block [22], [31]. This also prevented the use of more sophisticated phylogeny based calculations that can determine site or lineage specific dN/dS ratios [21], [23], [24].

Instead, examining polymorphisms site by site permitted the use of all the aligned sequence data without limiting the analysis to aligned sequence blocks. With this method, the 454 sequences and their associated polymorphisms were assumed to have diverged equally from the consensus sequence. The resulting dN/dS ratios characterize the diversity and provide a snap-shot of the diversification on-going in the environmental population. The phylogeny of the reads was not considered, therefore correcting for multiple substitutions (such as using the Jukes and Cantor correction) was not necessary. This approach also could not determine which polymorphisms were fixed in a population as the “population” was loosely defined based on genetic similarities which likely recruits sequence reads from multiple taxonomic levels.

### Defining the reads belonging to clades I and IV *Synechococcus* populations

As has been done previously [32], flow cytometry sorting was used to enrich for *Synechococcus* cells prior to sequencing, but as before the enrichment was not sufficient to assemble entire genomes or very large contigs from the metagenomes for population analysis. However, we were able to use the complete genomes of *Synechococcus* strains belonging to the dominant clades in the environment as scaffolds to collect and align sequences representing *Synechococcus* populations.

Pooling the metagenomes together, a total of 2 856 850 sequence reads equivalent to approximately 530 Mb were tested for alignment to the *Synechococcus* sp. CC9311 and *Synechococcus* sp. CC9902 genomes representing clades I and IV, respectively. The reads were tiled to both genomes simultaneously so that, if a read could align to both genomes with the given identity and coverage criteria, the better alignment to one of the two genomes was assigned. After tiling, two sets of sequence reads were obtained, one set aligning to CC9311, the other to CC9902.

**Figure 2:**
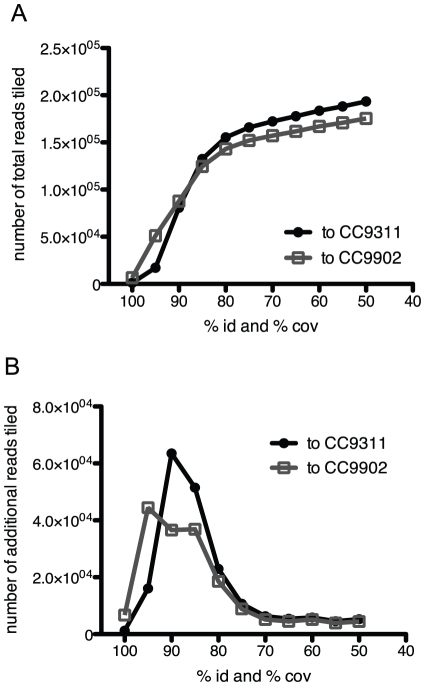
Number of metagenome sequence reads tiled to the CC9311 and CC9902 genomes. Total (A) and additional (B) metagenome sequence reads tiled to the CC9311 and CC9902 genomes as the % identity (id) and % coverage (cov) was lowered.

Tilings were tested with varying read and identity criteria. As the % identity and % coverage was lowered from 100 to 80%, a higher rate of sequence reads were recruited to the reference genomes (Figure 2). Below 80% identity and 80% coverage, the number of additional sequence reads recruited to the genomes drastically decreased. This suggests that the metagenome sequences were not uniformly divergent and there is likely a biological reason for this observed pattern of read recruitment.

The change in sequence recruitment at 80% identity and 80% coverage likely represents a threshold of genetic diversity that can delimit a taxonomic boundary within *Synechococcus*, specifically below clade-level. The criteria of 80% identity and 80% coverage was used to assemble population diversity for all subsequent analyses. Using these criteria, 138 734 (4.9% of the total number of sequence reads) and 131 261 (4.6%) reads tiled to the CC9311 and CC9902 genomes, respectively. 543 of the recruited reads hit both genomes equally. Most of these reads hit ribosomal RNA genes (85.3%) or other highly conserved genes and their effect on subsequent dN/dS calculations were negligible.

Some of the remaining metagenomic reads that did not tile to CC9311 and CC9902 under the criteria of 80% identity and 80% coverage likely also belong to *Synechococcus* from clades I and IV, but they are from genes that are not part of the CC9311 and CC9902 reference genomes or the sequences are less than 80% similar from the reference gene. For example, 13 002 reads tiled to the BL107 genome, another *Synechococcus* clade IV strain, that did not tile to CC9311 or CC9902. 25% of these reads tiled to genes only found in BL107. In addition, assembled contigs from the environmental metagenomes contained novel genes clearly belonging to clades I and IV (Palenik et al. unpublished data). For more divergent genes, the criteria of 80% identity and 80% coverage may be too strict to fully recruit reads belonging to clade I and IV *Synechococcus* and some diversity would be missed in the dN/dS calculation. To evaluate this, dN/dS ratios were also calculated with tiling using criteria of 70% identity and 70% coverage (see below).

### Read coverage and genomic variation

**Figure 3:**
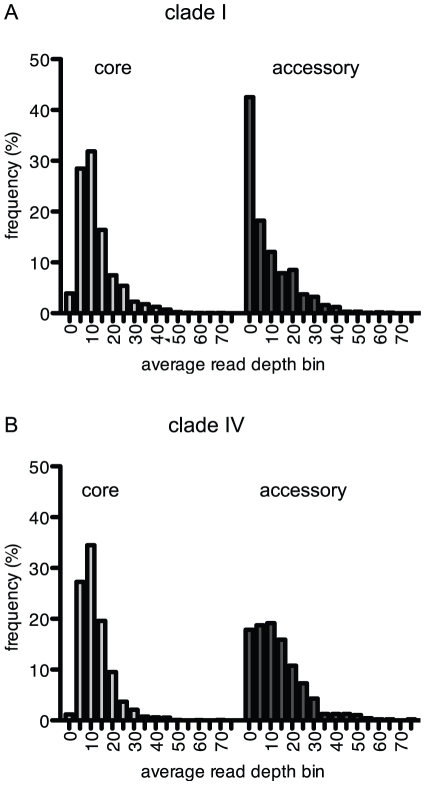
Histograms of the average depth of read coverage for core and accessory genes. The average depth of read coverage was calculated from tiling metagenome sequences to the CC9311 (A) and CC9902 (B) reference genomes. The tilings used criteria of 80% identity and 80% coverage.

The average depth of read coverage per gene varied ranging from from 0 to 68.7 and 72.6 for tilings to the CC9311 and CC9902 genomes, respectively with means of 11.1 and 12.3 (Figure 3). Read coverage was generally higher for core genes (i.e. orthologous genes found commonly in *Synechococcus* strains CC9311, CC9902, CC9605, and WH8102) than for accessory genes (i.e. genes that are found in some but not all sequenced genomes of *Synechococcus* strains). Because accessory genes are not found in all *Synechococcus* strains, their occurrence in natural populations was expected to be more rare than for the core genes. Metagenome reads covered a greater percentage of core genes by at least 5-fold (87.3 and 89.6% for the CC9311 and CC9902 tilings, respectively) than accessory genes (47.0 and 71.2). Accessory genes comprised the vast majority of genes with very low coverage (86.6 and 88.5% of the CC9311 and CC9902 genes, respectively with <2-fold average read coverage), but many accessory genes were not rare and were highly represented in the metagenomes (Tables S1 and S2). A greater percentage of CC9311 genes had less than 2-fold average depth of coverage than CC9902 (16.0 versus 3.8%) as CC9311 has a larger genome with a higher number of accessory genes some likely acquired through horizontal gene transfer [33].

### dN/dS and selection in the evolution of *Synechococcus*

After filtering the reads for sequencing errors, polymorphisms were identified for every nucleotide position in a gene as annotated in the reference genomes with at least 5-fold coverage of metagenome sequence reads (Tables S3 and S4). A minimum read coverage of 5-fold was used to include dN/dS calculations for genes that were more rare in the *Synechococcus* populations.

**Figure 4:**
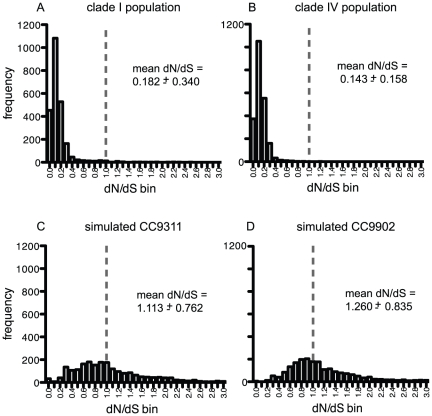
Histograms of dN/dS ratios. dN/dS ratios were calculated from gene alignments using metagenomic sequences from a *Synechococcus* clade I population (A), metagenomic sequences from a *Synechococcus* clade IV population (B), simulated CC9311 sequences (C) and simulated CC9902 sequences (D). The dashed line indicates where dN/dS = 1 and selection is neutral.

Based on the polymorphisms in the metagenome sequences tiled to the CC9311 and CC9902 genomes, the mean dN/dS was 0.182 and 0.143, respectively. The frequency distribution of the dN/dS ratios indicates that the large majority of genes (97 and 99%, respectively) have a dN/dS ratio <1 and have evolved under purifying selection (Figures 4A and B). Some sites of a gene may still have experienced positive selection, but the overall trend is purifying selection. Because core genes tend to be more conserved, as expected the mean dN/dS for core genes (0.114 and 0.119 for reads tiled to CC9311 and CC9902, respectively) was lower than for accessory genes (0.402 and 0.256).

The dN/dS ratios calculated using the tiling of 70% similarity and coverage were highly correlated to the tiling of 80% similarity and coverage (Figure S1). For the large majority of genes, the additional reads recruited to the clade I and IV populations using the less stringent criteria did not make a significant impact on the dN/dS ratios. Seven core genes and 50 accessory genes from the clade I population showed differences greater than 0.25 in their dN/dS ratios. While for the clade IV population, only 3 core genes and 7 accessory genes had dN/dS ratio differences greater than 0.25 (Tables S3 and S4). Eight genes (sync_1402, sync_1647, sync_1947, sync_2175, sync_2383, sync_2428, sync_2810, and sync_2912) with dN/dS ratios >1 using the 80% similarity and coverage recruitment, had their dN/dS ratios lowered to below or near 1 using the less stringent criteria.

A simulated 454 metagenome dataset was created comprising 300 000 sequences derived from the CC9311 and CC9902 genomes using MetaSim [34]. These sequences were generated to provide a null sample of reads of similar size to the actual metagenome reads that tiled to the CC9311 and CC9902 genomes. The simulation generated polymorphisms that occurred exclusively through random mutation introduced by 454 sequencing error resulting in mean dN/dS ratios of 1.113 and 1.260, respectively (Figures 4C and D). The dN/dS ratios were also more evenly distributed than the dN/dS ratios derived from natural populations indicating that the simulated mutations were not biased towards any form of selection. Therefore, the methods used here to calculate dN/dS accurately portrayed the simulated data as evolving neutrally without selection pressure.

dN/dS ratios for each gene were also calculated by averaging the pair-wise dN/dS ratios for each read aligned to a gene of the environmental consensus sequence (as calculated using PAML) (Tables S3 and S4). These resulting dN/dS ratios were significantly correlated with the dN/dS ratios calculated by identifying polymorphisms by site as described above (Figure S2). The pair-wise approach is commonly used to calculate dN/dS ratios for sequences in an alignment block. However, it is not ideally suited for environmental 454 metagenomes where sequence reads are aligned to varying locations along a gene and summarizing the data for a gene is not straightforward. Information was excluded because many of the aligned 454 reads were identical to the consensus sequence. This resulted in dN/dS ratios of 0/0 and these values could not be used in the mean dN/dS ratio calculated for the gene. In addition, reads that had only non-synonymous substitutions relative to the consensus sequence resulting in infinite dN/dS ratios (dN/0) were also excluded from the mean dN/dS calculated for the gene. Finally, read depth biased the results. Because the 454 reads were tiled in varying depths along the gene, the pair-wise dN/dS ratios from reads aligned to highly covered regions contributed more to the mean than the pair-wise dN/dS ratios from less covered regions.

### Environmental representation and dN/dS

**Figure 5:**
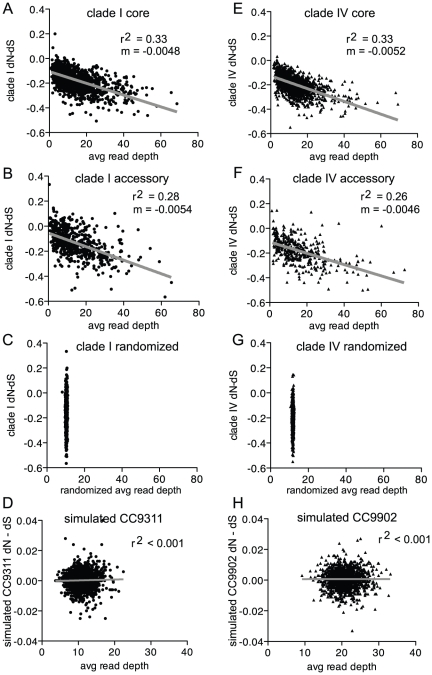
dN - dS versus the average depth of read coverage for each gene. For genes from the core genome of a clade I population (A), the accessory genome of a clade I population (B), randomized alignments of reads tiled to CC9311 (C), simulated CC9311 sequences (D), the core genome of a clade IV population (E), the accessory genome of a clade IV population (F), randomized alignments of reads tiled to CC9902 (G), and simulated CC9902 sequences (H). The gray line is the linear regression and m is the slope of the line. Note that dN - dS is plotted instead of dN/dS.

The depth of read coverage to a gene reflects the abundance or representation of this gene in the environmental samples. To examine the relationship of read coverage to dN/dS ratios, dN/dS ratios were compared to the average depth of coverage for each gene. There was a strong negative correlation between dN/dS and depth of coverage indicating that *Synechococcus* genes with high representation in the environment were more conserved evolutionarily. To better assess this correlation, the analysis was repeated using the difference dN - dS because ratio statistics (i.e. dN/dS) are susceptible to excessive variation when the denominator takes small values. Where dN - dS = 0 implies dN = dS and is therefore equivalent to the null hypothesis (no selection, dN/dS = 1). Again, a negative correlation was observed between read coverage and dN - dS (Figure 5). This relationship was not observed from a randomization of read alignment generating a null distribution of read depth per gene (Figure 5C and G) or from the simulated 454 read dataset (Figure 5D and H).

It was expected that core genes would be well covered by the metagenome sequences and be conserved with dN/dS ratios <<1 (or dN - dS<0). However, many accessory genes were also well represented by the metagenomes with low dN/dS ratios (Tables S1 and S2). The slopes of the linear regressions were only slightly different for the core (Figures 5A and E) compared to the accessory genes (Figures 4B and F). High sequence representation suggests that these genes are important in this environment regardless if they are from the core or accessory genomes, and the correlation with low dN - dS indicates that these genes are also evolutionarily conserved.

Most of the accessory genes with high sequence representation (considered to be greater than 20-fold coverage which is approximately the mean+1 standard deviation) that have evolved under purifying selection (low dN/dS) are poorly studied hypothetical genes with no known function (Tables S1 and S2). Some of these genes, however, are homologous to genes with very well characterized functions such as the CC9311 genes sync_0681 and sync_0682 that likely encode components of a ferrous iron transporter. These genes have atypical GC content and may have been horizontally transferred into an ancestor of CC9311 [35]. The high sequence representation and evolutionary conservation, however, points to the importance of these genes in the physiology and the ecological niches of *Synechococcus*. High sequence representation is likely due to the occurrence of the gene in a large proportion of the population, but may also be due to gene duplications, high rates of recombination or horizontal gene transfer, or the presence of phage genomes containing *Synechococcus* genes.

Several highly represented genes also may have evolved under positive selection as they have dN/dS ratios >1 (Tables 1 and 2). All of these genes are hypothetical with no known function (see below). Since they are abundant in the *Synechococus* population and are diversifying, these genes are likely important in the adaptation of *Synechococcus* to their environment.

**Table 1:** Selected genes with dN/dS ratios >1 based on polymorphisms from metagenomic sequences tiled to the CC9311 genome.

gene	description	dN/dS	dN/dS relative to CC9311	% of gene covered 5X/avg. read depth	core (C) or acce-ssory (A)	microarray gene expression changes	
						+	−
sync_1796	hypothetical	7.256	2.010	100/13.0	A	N	N
sync_0800	hypothetical	5.509	2.981	100/12.6	A	Y	N
sync_2079	hypothetical	3.818	1.364	21/2.8	A	N	Y
sync_1472	transcriptional regulator	2.121	2.429	4/0.9	A	Y	Y
sync_0523	conserved hypothetical	2.066	1.555	84/8.2	A	N	Y
sync_2833	hypothetical	2.046	3.161	100/14.9	A	N	Y
sync_2711	lipoprotein, putative	0.275	2.556	75/5.4	A	N	N
sync_0059	ribosomal protein L35	Inf	Inf	72/8.1	C	N	N
sync_0536	PsbM	Inf	Inf	43/4.4	C	N	Y
sync_0637	conserved hypothetical	Inf	Inf	0.5/0.5	A	N	Y
sync_0780	hypothetical	Inf	Inf	32/3.8	A	Y	N
sync_0838	conserved hypothetical	Inf	Inf	3/1.0	A	N	Y
sync_0889^#^	conserved hypothetical	Inf	Inf	26/3.7	C	N	Y
sync_0925	hypothetical	Inf	Inf	6/3.0	A	N	N
sync_1021	hypothetical	Inf	Inf	7/1.1	A	Y	N
sync_1490	ThiJ-like protein	Inf	Inf	29/2.9	A	Y	N
sync_1603	hypothetical	Inf	Inf	16/1.3	A	N	Y
sync_2225	hypothetical	Inf	Inf	16/1.4	A	Y	N
sync_2247	hypothetical	Inf	Inf	100/9.9	A	N	Y
sync_2506	hypothetical	Inf	Inf	17/3.7	A	Y	Y
sync_2513	hypothetical	Inf	Inf	19/2.4	A	Y	Y
sync_2649	conserved hypothetical	Inf	Inf	0.5/3.0	C	Y	N
sync_2902	hypothetical	Inf	Inf	98/7.9	A	N	Y

See Table S7 for complete list.

Inf indicates genes that had non-synonymous polymorphisms but no synonymous polymorphisms resulting in an infinite value for dN/dS.

**Table 2:** Genes with dN/dS ratios >1 based on polymorphisms from metagenomic sequences tiled to the CC9902 genome.

gene	description	dN/dS	dN/dS relative to CC9902	% of gene covered 5X/avg. read depth	core (C) or acce-ssory (A)	dN/dS CC9902 vs. BL107+
Syncc9902_1026	hypothetical	3.136	2.387	100/12.6	A	nh
Syncc9902_1174	hypothetical	2.933	2.933	100/22.3	A	nh
Syncc9902_1874	hypothetical	2.493	2.901	23/3.8	A	0.969
Syncc9902_1954	hypothetical	1.778	1.778	89/7.4	A	nh
Syncc9902_0240	conserved hypothetical	1.380	1.380	32/3.5	A	nh
Syncc9902_2008	hypothetical	1.329	1.244	100/37.1	A	1.275
Syncc9902_1801	conserved hypothetical	1.170	1.194	100/28.2	A	np
Syncc9902_1875	conserved hypothetical	1.168	1.197	96/7.9	A	1.939
Syncc9902_0450	hypothetical	1.099	1.063	100/60.2	A	1.562
Syncc9902_2037	conserved hypothetical	1.005	1.005	5/1.7	A	nh
Syncc9902_0923	Conserved hypothetical	Inf	Inf	20/3.4	C	0.119
Syncc9902_1576	Methylated-DNA-(protein)-cysteine S-methyltransferase	Inf	Inf	9/3.5	C	0.108
Syncc9902_2149	hypothetical	Inf	Inf	8/2.0	A	nh
Syncc9902_2225	hypothetical	Inf	Inf	4/1.2	A	nh
Syncc9902_2241	amino acid permease family	Inf	Inf	15/2.0	A	nh
Syncc9902_0591	hypothetical	0.465	0.466	89/12.4	C	2.850
Syncc9902_0952	Conserved hypothetical	0.216	0.218	100/26.4	C	2.075
Syncc9902_1111	Peptidase S13, D-Ala- D-Ala carboxypeptidase	0.150	0.153	90/13.7	C	1.038
Syncc9902_1718	hypothetical	0.742	0.738	100/16.9	A	1.309

See Table S8 for additional notes.

+Far right column provides the dN/dS ratios between homologous genes from the CC992 and BL107 genomes. nh indicates that there is no homologue between the two genomes. np indicates that there were no polymorphisms between the homologues.

Rare genetic components may also have a role in defining the ecological niche of a population because they provide unique functions. Unfortunately, metagenomics is not well suited for investigating the biology of these genes. Obtaining increased read coverage of these components, especially from a diverse community, would require exponentially greater sequencing effort. These regions may be better analyzed by targeted sequencing efforts rather than through metagenomics.

### Positive selection in *Synechococcus* populations

From the clade I and clade IV populations, 71 and 15 genes, respectively, had dN/dS ratios >1 indicating that more non-synonymous mutations were observed than expected by chance. This suggests that the genes may have evolved under positive selection (Tables 1 and 2). The majority of these genes (93 and 88%) encode hypothetical proteins, often <100 amino acids that are part of the accessory genome with no known homologs. These genes are especially interesting as they may provide unique functions that differentiate them from other *Synechococcus* and clues to possible adaptive phenotypes. Many of the genes with dN/dS ratios >1 (41 and 56%), however, were covered poorly by metagenome reads (<50% of the gene was covered at least 5-fold). Because positive selection has important implications, these observations of possible positive selection should be investigated across the majority of the gene and regions of site-specific positive selection using phylogeny-based methods could also be identified.

The use of environmental metagenomics provided a larger resource of data from which to assess population diversity and detect positive selection than using the genomes of cultured strains alone. In a comparison of homologous genes between CC9902 and BL107 (both clade IV strains with genome sequences available), only 7 genes showed evidence of positive selection (Table 2). Three of these, syncc9902_0450, syncc9902_1875, and syncc9902_2008 had dN/dS ratios >1 for both the environmental population and in the CC9902 vs. BL107 comparison.

Only a few well-covered genes with dN/dS>1 have annotated functions (Tables 1 and 2). For example, only non-synonymous polymorphisms were observed for the ribosomal protein L35 (sync_0059) (Table 1): histidine (hydrophilic, positively charged) to glutamine (hydrophilic, neutral) and proline (hydrophobic neutral) to alanine (hydrophobic neutral). A putative lipoprotein (sync_2711) has a dN/dS<1, but relative to the CC9311 genome instead of the metagenome consensus sequence, the dN/dS was 2.556 (Table 1). Otherwise, with the exception of these two genes, the other annotated genes all suffer from low sequence coverage and the calculated dN/dS ratios are likely less reliable.

**Figure 6:**
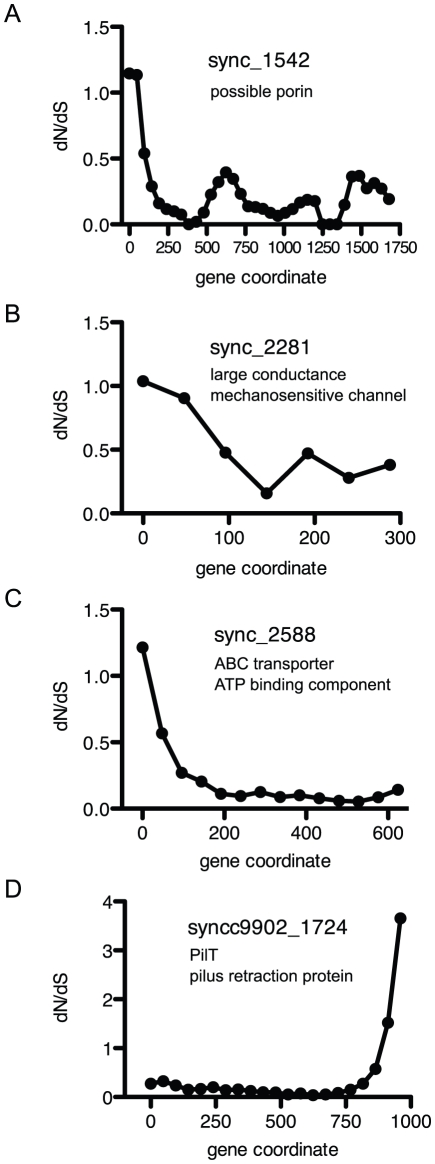
dN/dS ratios from sliding windows of 201 bp from genes with transport, secretory, or motility functions. These genes have regions of elevated dN/dS that were well-represented by metagenome reads.

dN/dS ratios were also calculated in sliding windows of 201 bp (67 aa) across each gene to examine potential regions of positive selection. 87 and 72 genes from the clade I and clade IV populations, respectively had regions of dN/dS>1 that were not observed when the dN/dS ratio was calculated over the entire gene (Tables S5 and S6). Of particular interest were several genes involved in transport, secretion, or motility functions that were well represented in the metagenome. Sync_1524, a possible porin gene, sync_2281, a large-conductance mechanosensitive channel, and sync_2588, the gene for a possible ABC transporter ATP-binding component all had elevated dN/dS at their N-termini (Figures 6A, B, and C). The C-terminus of syncc9902_1724, a homologue of PilT - the pilus retraction protein that functions in motility, had dN/dS>1 (Figure 6D).

### Assessing the function of hypothetical genes

As most of the genes with a dN/dS>1 (Tables 1 and 2) and highly covered accessory genes (Tables S1 and S2) are annotated as hypothetical, it is difficult to assess what functions they may provide or if in fact these are real genes. Different sources of information were used to determine if these hypothetical genes were annotated correctly and what their probable function might be. tBLASTn searches typically found no significant hit or the best hit was to hypothetical genes from other *Synechococcus* or *Prochlorococcus* genomes. Hits to other hypothetical genes at least indicate that these open reading frames are conserved within the *Synechococcus/Prochlorococcus* group, are likely true genes, and should be re-annotated as conserved hypothetical proteins.

Very few of the hypothetical genes had significant hits to protein fold databases. Only 13 (5.1%) of the hypothetical genes in Tables 1, 2, S1, and S2 had significant hits to known protein families (Table 3). Though far from definitive, these structural homologies do provide some clues as to the probable function of these genes. Hydropathy plots suggested that only one hypothetical protein, syncc9902_0591 might possibly have a transmembrane region. Interestingly, some hypothetical genes with dN/dS>1 are co-localized in the genome (eg. sync_1797 and sync_1796).

**Table 3:** Significant hits to protein superfamilies based on SCOP (Superfamily Classification of Proteins) of hypothetical genes with dN/dS>1 or hypothetical accessory genes with greater than 20-fold average read coverage.

gene locus	dN/dS	avg read coverage	SCOP identifier	Protein superfamily	e-value
sync_1132	0.100	26.3	55785	PYP-like sensor domain (PAS domain)	4.22×e^−10^
sync_1772	0.234	28.3	53474	alpha/beta hydrolases	6.10×e^−19^
sync_1952	0.147	31.0	53474	alpha/beta hydrolases	6.57×e^−24^
sync_2816	0.370	30.1	56112	protein kinase-like	1.09×e^−28^
sync_2497	0.257	34.0	103473	MFS general substrate transporter	2.52×e^−19^
sync_2817	0.429	49.0	53850	periplasmic binding protein-like II	5.03×e^−23^
syncc9902_0240	1.380	3.5	51182	RmlC-like cupins	4.44×e^−12^
syncc9902_0588	0.204	26.6	53850	periplasmic binding protein-like	3.78×e^−6^
syncc9902_1214	0.239	22.3	102405	MoCo carrier protein-like	6.74×e^−54^
syncc9902_1468	0.125	36.8	54909	dimeric alpha+beta barrel	5.31×e^−17^
syncc9902_2004	0.240	36.2	53254	phosphoglycerate mutase-like	6.51×e^−14^
syncc9902_2005	0.254	38.0	53474	alpha/beta hydrolases	7.11×e^−21^
syncc9902_2037	1.005	1.7	53756	UDP-glycosyltransferase/glycogen phosphorylase	1.06×e^−9^

Sync genes are from the CC9311 genome and syncc9902 genes are from the CC9902 genome.

Most of the CC9311 hypothetical genes showed differential expression in microarrays. Microarray data is available for CC9311 under stress conditions [36] (Palenik, Stuart, Tetu, Dupont, Johnson, Paulsen, et al. unpublished data). Under these conditions, 88% of the hypothetical genes with dN/dS ratios >1 showed differential expression with 44% of the genes increasing and 64% decreasing under one or more of the physiological shocks (Table 1). For the CC9311 hypothetical accessory genes with high metagenome coverage, 93% of the genes changed in expression level with 55% increasing and 57% decreasing (Table S1). This confirms that under certain physiological circumstances, mRNA corresponding to these hypothetical genes is expressed and that these are likely real genes. Genome-wide expression data is not available for CC9902 so a similar analysis could not be performed.

### dN/dS ratios of homologous genes

**Figure 7:**
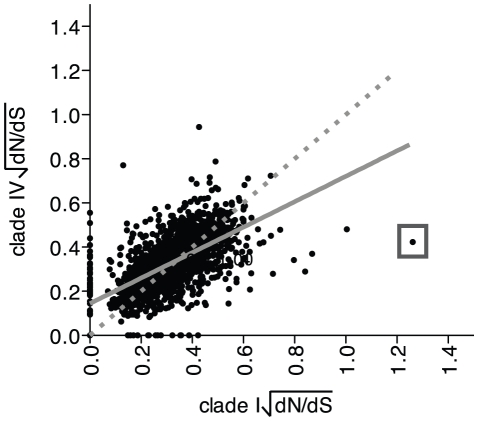
Scatter plot of dN/dS ratios of homologous genes from a clade IV population versus a clade I population. The dN/dS ratios were square root transformed prior to plotting to obtain a more normal distribution of values. The solid gray line is the linear regression (r^2^ = 0.38, y = 0.58x+0.14). The dotted line has a slope of 1. The boxed outlier corresponds to an ABC transporter for sugars, solute-binding protein (sync_1402 and syncc9902_1078). Sync_1402 was very poorly covered by metagenome reads (Table 1). Not plotted are 96 genes with no observed polymorphisms and 8 genes with no observed synonymous polymorphisms based on metagenome tilings to either the CC9311 or CC9902 genomes.

The dN/dS ratios from the clade I and IV populations were compared. For 2 054 homologous genes, the dN/dS ratios were similar in both populations (Figure 7). Based on a linear regression, the dN/dS ratio for each homologous gene was slightly higher in the clade IV population for ratios <0.35, and lower for ratios >0.35. Lower dN/dS ratios suggest that these genes experienced stronger purifying selection. However, dN/dS ratios can change over time and the rate of change is sensitive to population size and the “hitchhiking” of slightly deleterious mutations [37]. Thus, the comparison of dN/dS ratios between homologous genes from different populations is valid only if these parameters have been similar in the two populations since they diverged.

The vast majority of the homologous genes had dN/dS ratios <1. There were no genes with dN/dS ratios >1 in both clade I and IV populations. For 6 genes, the dN/dS ratios were >1 from the clade I population but <1 from clade IV. Four genes had a dN/dS ratio >1 from clade IV, but not from clade I (Tables 1 and 2, Figure 7). The genes with dN/dS ratios >1 tended to have low metagenome read coverage, so the estimates of dN/dS were not calculated over the whole gene. Sync_0059 (ribosomal protein L35) from the clade I population was the only homologous gene with good read coverage (72% of the gene covered at least 5-fold) and dN/dS >1. Sync_0523 and syncc9902_1875 (conserved hypothetical genes) are reciprocal best tBLASTn hits, both with dN/dS ratios >1 and good read coverage (Tables 1 and 2). However, syncc9902_1875 (251 amino acids) has a considerably larger open reading frame than sync_0523 (61 amino acids) and these may not be truly homologous genes. However, immediately upstream of sync_0523, a point mutation has clearly introduced a stop codon and the sequence continues to align with syncc9902_1875 for an additional 23 amino acids. This region of the CC9311 genome also contains a tRNA and other small hypothetical genes, so this could be a highly variable region of the genome with increased susceptibility to genome rearrangements, genetic transfers or deletions.

## Discussion

By developing a method to calculate dN/dS using 454 metagenome sequences, a snap-shot of the diversification occurring in marine *Synechococcus* populations was characterized. The majority of the genes from the two populations of *Synechococcus* were shown to have evolved under purifying selection and most of the observed polymorphisms were selectively neutral (or nearly neutral). By comparing genome sequences between strains of *Synechococcus* across different clades, at this scale of phylogeny, the dN/dS ratios of core genes were also well below 1 [19]. Purifying selection is also the dominant mode of selection in other microbial populations [17], [18].

Of the small percentage of genes with a greater number of non-synonymous polymorphisms than expected by chance (dN/dS>1) suggesting positive selection, most were hypothetical genes with no known function. Although previous studies have shown that positively selected genes typically encode proteins that are exposed on the cell surface [8], we were unable to determine possible structures or functions for most of the hypothetical genes undergoing positive selection. The gene for the ribosomal protein L35 (sync_0059) from the clade I population was the only well-covered, annotated gene with dN/dS>1. Positive selection has been shown for the circadian clock gene *kaiC* from the benthic cyanobacteria *Microcoleus chthonoplastes* and for *rbcX*, a possible RubisCo chaperone from the heterocystous cyanobacteria *Anabaena/Aphamizomenon* sp. [38]. However, for the two *Synechococcus* populations analyzed here, *kaiC* has evolved under purifying selection (Tables S3 and S4) and *rbcX* is not found in marine *Synechococcus*.

The ribosomal protein L35 is one of the smallest proteins associated with the large subunit (23S) of the ribosome. It is curious that a ribosomal protein, usually considered to be highly conserved proteins, would have a high dN/dS ratio. L35 has not been studied as intensively as other ribosomal proteins probably due to its small size, so the effects of the non-synonymous polymorphisms are not known [39]. L35 is part of a dicistronic operon with L20, also a protein of the ribosomal large subunit. The expression of L35 and L20 is auto-regulated by L20 which acts as a repressor protein that binds upstream of the L35 gene [40]. Previous evidence suggests some ribosomal proteins, including L20 but not L35, may have been transferred amongst the *Synechococcus/Prochlorococcus* group [41]. As L35 and L20 form an operon, if the ribosomal proteins were transferred separately, then L35 may be diversifying to adapt to L20 genes originating from a different genetic environment. L35 is also involved in ribosome assembly, so changes affecting the assembly process may also be under selection. In addition, some ribosomal proteins have evolved and been horizontally transferred based on their ability to bind zinc [42]. As histidines are often involved in metal binding and histidines were polymorphic in the L35 protein, metal bioavailability may be a driving factor in the evolution of this gene in *Synechococcus* populations.

Using a window of nucleotides to measure dN/dS, many more genes were identified with regions of dN/dS ratios >1 within the gene (Tables S5 and S6). These included hypothetical genes and genes with annotated functions, particularly potential transporters. A porin gene (sync_1542) with high dN/dS at its N-terminus, is homologous with a porin gene SYNW2224 from *Synechococcus* sp. WH8102 whose expression is induced under phosphate stress [43]. These porins are also homologous with Som A and B from *Synechococcus* sp. PCC6301. Som A and B are thought to be trimeric with signal peptides at their N-termini and their C-terminus has amino acids important for insertion into the outer membrane [44], [45]. Sync_1542 lacks detectable signal peptides at its N-terminus, so possible reasons for the elevated dN/dS at this region are not known.

Another interesting gene is syncc9902_1724, homologous to pilus retraction protein PilT, with elevated dN/dS at its C-terminus. PilT proteins are part of a larger family of type II secretion proteins. These all have a conserved core with NTPase function, but it is thought that the differing sequences at their N and C-termini confer more specific functions such as protein secretion, type IV pilus assembly or retraction, and natural competence [46]. Thus positive selection at the C-terminus of syncc9902_1724 may have direct functional consequences.

### Population diversity, genomic variation, and dN/dS

The population diversity within a clade of *Synechococcus* was defined by sequences with at least 80% identity and 80% coverage to the CC9311 (clade I) and CC9902 (clade IV) reference genomes. This is by no means a sharp boundary as some genes necessitate strong conservation and tolerate less variation than others. Thus, there may be sequences with greater than 80% identity that do not belong to these particular *Synechococcus* clades and vice versa. However, it is unlikely that sequences tiled to the reference genomes do not actually belong to clades I or IV because *Synechococcus* from clades other than I and IV were rare in this environment [26].

It is currently impossible to analyze the complete population diversity of clades I and IV as the list of accessory genes (genes found in some but not all members of the clade) continues to grow. The dN/dS analysis was limited to the reads tiled to one reference genome from each of the dominant clades in the Southern California Bight environment. These strains (CC9311 from clade I and CC9902 from clade IV) were appropriate representatives as they were from isolated from this region [47].

In addition, read coverage per gene varied because the environmental populations of *Synechococcus* were not uniform in gene content. The *Synechococcus* community in the Southern California Bight is dominated by clades I and IV, but tremendous diversity exists within these clades. Comparative genomics and genome hybridizations have demonstrated that strains within a clade do not share the same genetic make-up ([33]; Tai, Paulsen, Palenik et al. unpublished data). Thus, genes from the reference genomes that were rare in the environmental populations would have low sequence representation in the metagenomes. In contrast, genes with high read coverage were likely common components of *Synechococcus* genomes in the environment and therefore were sequenced more frequently. Genes that may have been duplicated or in greater copy number in environmental genomes compared to the reference genomes would also have resulted in relatively higher sequence representation. Although the relationship between sequence abundance and environmental abundance is likely skewed due to biases in whole genome amplification and 454 sequencing, these biases tend to be random or without a simple, known explanation [48]–[51]. Interestingly, we nevertheless found that genes with greater sequence representation are more conserved with lower dN/dS ratios (Figure 4).

### Purifying selection and population size

The predominance of purifying selection in *Synechococcus* populations likely reflects the efficiency of purifying selection for slightly deleterious mutations when the effective population size (N_e_) is large [52]. For *Synechococcus*, the assumption that N_e_ is large is thought to be appropriate given the vast number of these microorganisms in the coastal Southern California Bight. Both clade I and clade IV *Synechococcus* can reach abundances of 10^5^/ml [26]. Given a conservative estimate of 10^3^/ml over the entire region (∼170 000 km^2^), their population sizes are on the order of 10^16^. But how the census population size compares to N_e_ is not clear [53].

Many aspects of microbial population genetics, especially of environmental populations are very poorly understood, including the mutation rate, the selection coefficients on different types of mutations, the rate of migration, and N_e_ for different microbial populations [54]. Even the ecological and taxonomic scales that define a microbial population and thus N_e_ are controversial [55]–[57]. In addition, the combined effect of generation time, mutation rate, and rate of environmental change on the diversity of a population are not clearly understood [58]. These parameters will be necessary to obtain a more accurate portrayal of the forces that shape microbial population diversity and evolution.

### Conclusions

Population genetics provides a theoretical framework to understand diversification, adaptation, and evolution. While the population genetics of microorganisms has long been studied experimentally with cultured isolates, environmental metagenomics provides a tremendous resource of data from native microbial communities that is ripe for microbial population genetics analysis. To make use of these new sources of data, new analytical methodologies are required.

By developing methods to calculate dN/dS ratios from Roche 454 sequence reads, the predominance of purifying selection was demonstrated from environmental metagenomes of two co-existing coastal *Synechococcus* populations. Genes showing evidence of positive selection were mostly hypothetical genes but are likely true genes because they are expressed and conserved among different *Synechococcus* strains. The analysis described here provided the first insight into the role of selection in the evolution of *Synechococcus*, a globally significant primary producer.

As more metagenomes become available, more opportunities arise to investigate the factors that have given rise to the observed diversity. Further methods are in need of development, however, to take advantage of these data and derive fundamental evolutionary parameters, such as mutation, recombination, and migration rates, for understanding microbial evolution. Hopefully, as these analytical challenges are met, we will gain a better understanding of the ecological and evolutionary scales under which microbes adapt.

## Materials and Methods

### Environmental sampling, sorting, and sequencing

Four samples were collected from surface waters at the end of the Scripps Institution of Oceanography (SIO) pier, La Jolla, CA (32.8672 N, 117.2583 W). As previously described for a metagenomic sample from October 10, 2006 [32], samples were similarly collected and sorted on May 17, 2007, March 6, 2008, and April 17, 2008. On May 15, 2008, samples were collected from the surface and at 20 m depth using a niskin bottle from a station approximately 1.7 km off-shore of the SIO pier (32.8733 N, 117.2750 W).

For each sample, approximately 10 liters of seawater were pre-filtered through a 2 µm polycarbonate filter (GE Osmonics) then the microorganisms were collected onto a 0.2 µm Supor filter disc (Pall). The microorganisms were resuspended from the filter by vortexing in approximately 10 ml of 0.2 µm filtered seawater.

*Synechococcus* were enriched using flow cytometry sorting and collected onto a 0.2 µm Supor filter disc and stored at −80°C as previously described [32]. DNA was extracted, amplified by multiple displacement amplification (MDA) using phi 29 DNA polymerase from the Genomiphi v2 kit (GE Healthcare), and sequenced as previously described [32] except using a GS-FLX instrument (454 Sequencing, Roche) providing reads averaging 250 bp in length. The sequences previously published from 10/10/06 averaged 100 bp in length [32] and were also included in this study. Sequences have been deposited at NCBI as Project ID 66351.

### Sequence tiling to CC9311 and CC9902 genomes

The metagenomic sequences from the 6 samples were pooled and analyzed together. Using the Reference Assembly tool from the CLC Genomics Workbench (version 3.2), the pooled environmental metagenomic sequences were tiled (a.k.a. aligned, recruited) to the *Synechococcus* CC9311 (clade I) and *Synechococcus* CC9902 (clade IV) genomes simultaneously. These genome sequences represent the two dominant *Synechococcus* clades in the coastal Southern California Bight and tilings to *Synechococcus* genomes from other clades resulted in very poor sequence recruitment ([32]; Palenik, Ren, Tai, Paulsen unpublished data).

Different tiling parameters were tested. The % identity and % coverage (length fraction) were varied between 100 and 50%, while using alignment penalties of mismatch = 1, insertion = 2, and deletion = 2. For each tiling, two alignments resulted comprising separate, non-overlapping sets of metagenome sequences, one with sequence reads aligned to the CC9311 genome and another to the CC9902 genome. As the reads were tiled to the CC9311 and CC9902 genomes simultaneously, reads that would have hit both genomes with the given parameters were recruited to the genome with the best match. Equal hits were assigned randomly. The number of new sequences tiled to the reference genomes decreased as the % identity parameter was lowered below 80% (Figure 2). Thus the tiling resulting from 80% identity and 80% coverage was used for all subsequent analyses.

To confirm that the reads tiling to the CC9311 and CC9902 genomes with 80% identity and 80% coverage encompassed a *Synechococcus* clade I or IV population, a BLASTn search of the non-redundant nucleotide database from GenBank was performed [59]. For all reads that tiled to either the CC9311 or CC9902 genomes, the best BLASTn hit was determined. It was expected that reads tiling to the CC9311 genome would have this genome as the top hit and similarly with the CC9902 tiling. Of the reads recruited to the CC9311 genome, 96.9% had the CC9311 genome as their top BLASTn hit, 2.4% had environmental sequences likely from clade I *Synechococcus*, 0.6% had other *Synechococcus* or *Prochlorococcus* strains, 0.08% had other bacteria, and 0.05% had cyanophages. Similarly 97.2% of the reads tiling to CC9902 also had CC9902 as their top BLASTn hit, 1.9% had environmental sequences likely from clade IV *Synechococcus*, 0.8% had other *Synechococcus* or *Prochlorococcus* strains, 0.07% had other bacteria, and 0.04% had cyanophages.

### Analysis of tiled reads and dN/dS calculation

The CC9311 and CC9902 alignments resulting from the tiling using the criteria of 80% identity and 80% coverage were exported from the CLC Genomics Workbench. Custom Python scripts (available upon request) were implemented to analyze the alignments. For each nucleotide position of the reference genomes, the depth of read coverage and the occurrence of polymorphisms were recorded (Figure 1). Although sequences derived using 454 technology are greater than 99.9% accurate [60], [61], strict quality criteria were used to remove poorly called nucleotides from the sequence alignments. Nucleotides from the sequence reads were only considered if they had a quality score above 20 and the average quality score of the surrounding 11 nucleotides was greater than 15. A nucleotide position was considered polymorphic if the aligned nucleotides were not identical (but not considering Ns). Insertions or deletions relative to the reference genomes were not considered.

A majority-rule consensus sequence was determined from the metagenome reads aligning to the reference genomes. The gene annotations from the reference genomes were used to define the start and stop coordinates of genes in the consensus sequences. For each gene, dN/dS was calculated as:
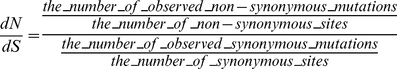
The consensus sequence was used to calculate the number of non-synonymous and synonymous sites for each nucleotide of a gene. A site is a possible change (mutation) for each nucleotide position of a codon. If the possible mutation to the codon would result in an amino acid change, this is a non-synonymous site. If there is no amino acid change, it is a synonymous site. Each possible mutation was considered to determine the number of sites. For each observed polymorphic nucleotide position, if the alignment contained a nucleotide that differed from the consensus, the type of mutation (non-synonymous or synonymous) was determined. The type of mutation was counted only for positions that were covered by a depth of at least 5 metagenomic sequence reads.

dN/dS ratios were also determined across a gene in a window of 201 nucleotides in steps of 48 nucleotides.

The polymorphisms and the consensus sequences were derived solely from the environmental *Synechococcus* community because they were determined from the metagenome sequences only and the reference genome sequences were excluded.

dN/dS ratios were also calculated using the reference genome (i.e. CC9311 or CC9902) instead of the consensus sequence as the basis for determining whether the polymorphisms were synonymous or non-synonymous.

dN/dS ratios between homologous genes of CC9902 and *Synechococcus* sp. BL107 (GenBank accession NZ_AATZ00000000) were also determined. BL107 open reading frames (ORFs) were aligned to the CC9902 genome using the Reference Assembly tool of the CLC Genomics Workbench with parameters of 80% identity, 80% coverage, and alignment penalties of mismatch = 1, insertion = 3, and deletion = 3. dN/dS ratios from the exported alignment were calculated as described above.

### Pair-wise dN/dS using PAML

As a comparison, for each read aligned to the consensus sequence, dN/dS values were determined using the yn00 method implemented in PAML [31], [62]. 454 reads are not usually long enough to cover an entire gene sequence and the reads are aligned to different sections of a gene. So to obtain a dN/dS estimate for a gene, the pair-wise dN/dS ratios for each read aligned to a gene were averaged, but excluding all dN/dS values of 0/0 (not a number) and x/0 (infinity).

### Analysis of hypothetical genes with high read coverage and dN/dS>1

Bioinformatics tools were used to assess possible functions of hypothetical genes and experimental data was examined to determine if hypothetical genes are expressed. A tBLASTn search of the non-redundant GenBank database was used to find the best hit to the hypothetical genes [59]. Note that genes from *Synechococcus* sp. BL107, a strain belonging to the same clade as CC9902, are not currently included in the non-redundant GenBank database. Hits to genes with a low e-value provided evidence that the gene is real and annotated correctly. For microarray expression data existing for CC9311 under stress conditions, i.e. exposed to toxins (ethidium bromide and mitomycin C), salt shock, and copper shocks [36] (Palenik, Stuart, Tetu, Dupont, Johnson, Paulsen et al. unpublished data), it was noted if these genes showed significant changes in expression. Possible structures for these genes were determined using the Superfamily SCOP classification (http://supfam.cs.bris.ac.uk/SUPERFAMILY_1.73/hmm/html) [63]. The results were corroborated by using HHpred (http://toolkit.tuebingen.mpg.de/hhpred) [64]. Hydropathy plots were examined to determine if any of the hypothetical proteins might have transmembrane regions. The hydrophobic character of the protein was determined using the method of Kyte and Doolittle [65] using a window size of 19 amino acids (http://www.vivo.colostate.edu/molkit/hydropathy/index.html).

### Sequence simulations and randomization tests

Simulated metagenome sequences were generated to test analytical methods. 300 000 454-like sequence reads were simulated from the CC9311 and CC9902 genomes using MetaSim (http://www-ab.informatik.uni-tuebingen.de/software/metasim) [34]. MetaSim introduces systematic sequencing errors known to occur from 454 sequencing into the simulated reads. These sequences were tiled to the reference genomes and analyzed as for the actual 454 sequence reads from the environment.

To assess the significance of the varying depth of read coverage per gene, randomized alignments of 454 reads were generated. A null distribution of read coverage was generated by randomly assigning the location where the 454 reads tiled to the reference genomes. This was repeated 100 times and a mean depth of coverage for each gene for the 100 randomizations was calculated.

### Statistical analyses

All statistical analyses were conducted with Prism (version 5.0b, GraphPad Software Inc.). To compare dN/dS ratios from the clade I and IV populations, the dN/dS ratios were square-root transformed to obtain a more normal distribution of values.

## Supporting Information

Figure S1 **Pearson correlation of dN/dS ratios calculated using reads tiled using criteria of 70% similarity and 70% coverage (70/70) vs. 80% similarity and 80% coverage.** A) clade I population. B) clade IV population.
(EPS)

Figure S2 **Spearman correlation of dN/dS ratios calculated from pair-wise alignments using PAML versus calculation by site.** A) clade I population. B) clade IV population.
(EPS)

Table S1 **dN/dS ratios based on metagenomic sequences tiled to the CC9311 genome for accessory genes with greater than 20-fold average depth of read coverage.** 20-fold is approximately 1 standard deviation greater than the mean. The percentage of the gene covered by at least 5-fold read coverage (%min5Xcov), the average depth of read coverage (avg read depth), and whether the gene changed in expression level under stress conditions assessed by microarray analysis are also shown.
(XLS)

Table S2 **dN/dS ratios based on metagenomic sequences tiled to the CC9902 genome for accessory genes with greater than 20-fold average depth of read coverage.** 20-fold is approximately 1 standard deviation greater than the mean. The percentage of the gene covered by at least 5-fold read coverage (%min5Xcov) and the average depth of read coverage (avg read depth) are also shown.
(XLS)

Table S3 **dN/dS ratios based on metagenomic reads tiled to the CC9311 genome with 80% similarity and 80% coverage.** dN, dS, the percentage of the gene covered by at least 5-fold read coverage (%min5Xcov) and 1-fold read coverage (%min1Xcov), the average depth of read coverage (avg read depth), dN/dS calculated relative to the CC9311 genome sequence rather than a population consensus sequence (dN/dS vs CC9311), dN/dS calculated using a metagenome tiling of 70% similarity and 70% coverage (dN/dS 70_70), and pair-wise dN/dS calculated using PAML are also shown. For all of the dN/dS columns except for the PAML analysis, NaN indicates a dN/dS of 0/0. Inf indicates that only non-synonymous polymorphisms were detected. For the PAML analysis, reads that had only non-synonymous polymorphisms relative to the consensus (dN/dS = infinite) were not included. Accessory genes are highlighted in blue.
(XLS)

Table S4 **dN/dS ratios based on metagenomic reads tiled to the CC9902 genome with 80% similarity and 80% coverage.** dN, dS, the percentage of the gene covered by at least 5-fold read coverage (%min5Xcov) and 1-fold read coverage (%min1Xcov), the average depth of read coverage (avg read depth), dN/dS calculated relative to the CC9902 genome sequence rather than a population consensus sequence (dN/dS vs CC9902), dN/dS calculated with the metagenome tiled with 70% similarity and 70% coverage (dN/dS 70_70), and pair-wise dN/dS calculated using PAML are also shown. For all of the dN/dS columns except for the PAML analysis, NaN indicates a dN/dS of 0/0. Inf indicates that only non-synonymous polymorphisms were detected. For the PAML analysis, reads that had only non-synonymous polymorphisms relative to the consensus (dN/dS = infinite) were not included. Accessory genes are highlighted in blue.
(XLS)

Table S5 **dN/dS ratios from 201 bp sliding window of metagenomic reads tiled to the CC9311 genome.** The position of the window start and end coordinates based on the CC9311 genome are provided. Inf indicates that only non-synonymous polymorphisms were observed. * genes with dN/dS>1 for the entire gene.
(XLS)

Table S6 **dN/dS ratios from 201 bp sliding window of metagenomic reads tiled to the CC9902 genome.** The position of the window start and end coordinates based on the CC9902 genome are provided. Inf indicates that only non-synonymous polymorphisms were observed. * genes with dN/dS>1 for the entire gene.
(XLS)

Table S7 **Complete list of genes with dN/dS ratios >1 based on polymorphisms from metagenomic sequences tiled to the CC9311 genome.**
(DOCX)

Table S8 **Genes with dN/dS ratios >1 based on polymorphisms from metagenomic sequences tiled to the CC9902 genome.**
(DOCX)
