# Supplementary material for: Selection in Coastal Synechococcus (Cyanobacteria) Populations Evaluated from Environmental Metagenomes
Source: PLoS One. 2011 Sep 9;6(9):e24249. doi: 10.1371/journal.pone.0024249 (PMC3170327; doi:10.1371/journal.pone.0024249)
Supplement: Table S7 — Complete list of genes with dN/dS ratios >1 based on polymorphisms from metagenomic sequences tiled to the CC9311 genome. (DOCX) [file pone.0024249.s009.docx]

Table S7. Genes with dN/dS ratios > 1 based on polymorphisms from metagenomic sequences tiled to the CC9311 genome.

| gene | description | dN/dS | dN/dS relative to CC9311 | % of gene covered 5X /avg. read depth | core (C) or accessory (A) | best tBLASTn hit | microarray gene expression changes | |
| --- | --- | --- | --- | --- | --- | --- | --- | --- |
|  |  |  |  |  |  |  | increased | decreased |
| sync_1796 | hypothetical protein | 7.256 | 2.010 | 100/13.0 | A | ns | N | N |
| sync_0800 | hypothetical protein | 5.509 | 2.981 | 100/12.6 | A | ns | Y | N |
| sync_2079 | hypothetical protein | 3.818 | 1.364 | 21/2.8 | A | ns | N | Y |
| sync_1306 | hypothetical protein | 3.678 | 5.884 | 100/11.4 | A | ns | N | Y |
| sync_1085 | hypothetical protein | 3.428 | 2.368 | 42/4.5 | A | ns | N | N |
| sync_1782 | hypothetical protein | 3.235 | 4.079 | 100/35.3 | A | ns | N | Y |
| sync_0585 | hypothetical protein | 2.752 | 1.174 | 28/4.0 | A | ns | N | Y |
| sync_1828 | hypothetical protein | 2.718 | 2.044 | 74/8.6 | A | ns | N | N |
| sync_2359 | hypothetical protein | 2.505 | 1.089 | 81/5.4 | A | ns | N | Y |
| sync_2115 | hypothetical protein | 2.474 | 2.474 | 100/18.4 | A | ns | Y | Y |
| sync_1784 | hypothetical protein | 2.290 | 1.442 | 93/6.7 | A | ns | Y | N |
| sync_2273 | hypothetical protein | 2.259 | 2.198 | 100/17.0 | A | ns | N | Y |
| sync_0324 | hypothetical protein | 2.225 | 2.309 | 30/3.8 | A | ns | Y | Y |
| sync_1348 | hypothetical protein | 2.199 | 1.753 | 100/11.8 | A | ns | Y | Y |
| sync_2834 | hypothetical protein | 2.161 | 1.248 | 57/5.2 | A | ns | N | Y |
| sync_1472 | possible transcriptional regulator protein | 2.121 | 2.429 | 4/0.9 | A | *Prochlorococcus* MIT9313 | Y | Y |
| sync_0523^ | conserved hypothetical protein* | 2.066 | 1.555 | 84/8.2 | A | *Synechococcus* CC9902 | N | Y |
| sync_2833 | hypothetical protein | 2.046 | 3.161 | 100/14.9 | A | ns | N | Y |
| sync_1626 | hypothetical protein | 1.944 | 2.208 | 55/3.9 | A | ns | N | Y |
| sync_0552 | hypothetical protein | 1.934 | 1.934 | 44/4.2 | A | ns | Y | N |
| sync_1761 | hypothetical protein | 1.853 | 1.367 | 93/10.8 | A | ns | Y | N |
| sync_1047 | hypothetical protein | 1.800 | 1.232 | 63/6.7 | A | ns | N | Y |
| sync_0588 | hypothetical protein | 1.774 | 1.398 | 100/7.8 | A | ns | N | Y |
| sync_2178 | hypothetical protein | 1.739 | 1.421 | 100/15.2 | A | ns | N | Y |
| sync_1791 | hypothetical protein | 1.678 | 1.803 | 100/18.9 | A | ns | Y | Y |
| sync_1947 | hypothetical protein | 1.650 | 1.650 | 11/2.7 | A | ns | N | Y |
| sync_1402^#^ | ABC transporter for sugars, solute binding protein | 1.591 | 1.512 | 2/1.9 | C | *Synechococcus* WH7803 | N | Y |
| sync_2076 | hypothetical protein | 1.578 | 1.387 | 82/23.4 | A | ns | Y | Y |
| sync_2428 | hypothetical protein | 1.526 | 1.255 | 98/7.6 | A | ns | N | Y |
| sync_2912 | hypothetical protein | 1.514 | 1.333 | 13/2.3 | A | ns | N | Y |
| sync_1049 | hypothetical protein | 1.468 | 1.188 | 68/8.1 | A | ns | N | N |
| sync_1859 | hypothetical protein | 1.429 | 1.527 | 100/18.5 | A | ns | N | N |
| sync_2186 | hypothetical protein | 1.314 | 0.678 | 100/6.5 | A | ns | N | Y |
| sync_1647 | hypothetical protein | 1.296 | 0.789 | 33/3.7 | A | ns | Y | N |
| sync_0864 | hypothetical protein | 1.273 | 1.574 | 38/7.2 | A | ns | N | Y |
| sync_1797 | hypothetical protein | 1.258 | 1.406 | 33/4.0 | A | ns | N | Y |
| sync_0645 | hypothetical protein | 1.219 | 1.172 | 81/9.1 | A | ns | Y | N |
| sync_2383 | hypothetical protein | 1.207 | 0.703 | 22/1.8 | A | ns | Y | Y |
| sync_2810 | hypothetical protein | 1.195 | 1.540 | 81/9.7 | A | ns | N | Y |
| sync_2175 | hypothetical protein | 1.179 | 1.300 | 47/2.9 | A | ns | N | Y |
| sync_2944 | hypothetical protein | 1.167 | 1.109 | 45/3.4 | A | ns | N | Y |
| sync_1128 | hypothetical protein | 1.161 | 1.158 | 38/17.6 | A | ns | Y | Y |
| sync_1648 | hypothetical protein | 1.159 | 1.367 | 100/11.0 | A | ns | Y | N |
| sync_1519 | Breast cancer type 1 susceptibility protein homolog | 1.152 | 1.193 | 40/4.4 | A | Uncultured cyanobacterium | N | N |
| sync_2849 | hypothetical protein | 1.110 | 1.581 | 57/4.2 | A | ns | N | Y |
| sync_1822 | hypothetical protein | 1.102 | 1.236 | 90/8.1 | A | ns | Y | N |
| sync_0951 | hypothetical protein | 1.046 | 1.056 | 100/21.7 | A | ns | N | Y |
| sync_1481 | hypothetical protein | 1.043 | 0.720 | 6/1.0 | A | ns | Y | N |
| sync_2435 | conserved hypothetical protein* | 1.031 | 0.934 | 39/3.6 | A | *Prochorococcus* MIT9313 | Y | N |
| sync_1155 | hypothetical protein | 1.021 | 1.172 | 100/25.8 | A | ns | Y | Y |
| sync_1520 | hypothetical protein | 1.014 | 0.712 | 19/3.4 | A | ns | Y | N |
| sync_0708^#^ | hypothetical protein | 1.008 | 1.050 | 71/8.3 | C | *Synechococcus* WH7803 | N | N |
| sync_2406 | hypothetical protein | 1.003 | 0.868 | 80/10.7 | A | ns | Y | N |
| sync_2590 | hypothetical protein | 0.980 | 1.004 | 100/15.6 | A | ns | Y | N |
| sync_1778 | hypothetical protein | 0.881 | 1.013 | 100/47.7 | A | ns | N | Y |
| sync_1235 | hypothetical protein | 0.785 | 1.034 | 37/3.7 | A | *Synechococcus* CC9902 | Y | N |
| sync_1164 | hypothetical protein | 0.975 | 1.181 | 82/6.9 | A | ns | Y | N |
| sync_2070 | hypothetical protein | 0.763 | 1.571 | 87/10.6 | A | ns | Y | Y |
| sync_1255 | hypothetical protein | 0.734 | 1.651 | 53/5.6 | A | ns | N | Y |
| sync_2477 | hypothetical protein | 0.890 | 1.776 | 64/4.2 | A | ns | Y | Y |
| sync_2711 | lipoprotein, putative | 0.275 | 2.556 | 75/5.4 | A | ns | N | N |
| sync_0059^#^ | ribosomal protein L35 | Inf | Inf | 72/8.1 | C | *Synechococcus* CC9605 | N | N |
| sync_0536^#^ | photosystem II reaction center protein PsbM | Inf | Inf | 43/4.4 | C | *Synechococcus* WH7803 | N | Y |
| sync_0637 | conserved hypothetical protein* | Inf | Inf | 0.5/0.5 | A | *Prochorococcus* MIT9313 | N | Y |
| sync_0780 | hypothetical protein | Inf | Inf | 32/3.8 | A | ns | Y | N |
| sync_0838 | conserved hypothetical protein* | Inf | Inf | 3/1.0 | A | *Synechococcus* CC9605 | N | Y |
| sync_0889^#^ | conserved hypothetical protein* | Inf | Inf | 26/3.7 | C | *Synechococcus* CC9605 | N | Y |
| sync_0925 | hypothetical protein | Inf | Inf | 6/3.0 | A | ns | N | N |
| sync_1021 | hypothetical protein | Inf | Inf | 7/1.1 | A | ns | Y | N |
| sync_1490 | ThiJ-like protein | Inf | Inf | 29/2.9 | A | Picea sitchensis | Y | N |
| sync_1603 | hypothetical protein | Inf | Inf | 16/1.3 | A | ns | N | Y |
| sync_2225 | hypothetical protein | Inf | Inf | 16/1.4 | A | ns | Y | N |
| sync_2247 | hypothetical protein | Inf | Inf | 100/9.9 | A | ns | N | Y |
| sync_2506 | hypothetical protein | Inf | Inf | 17/3.7 | A | ns | Y | Y |
| sync_2513 | hypothetical protein | Inf | Inf | 19/2.4 | A | ns | Y | Y |
| sync_2649^#^ | conserved hypothetical protein* | Inf | Inf | 0.5/3.0 | C | *Synechococcus* WH8102 | Y | N |
| sync_2902 | hypothetical protein | Inf | Inf | 98/7.9 | A | ns | N | Y |

Inf indicates genes that had non-synonymous polymorphisms but no synonymous polymorphisms resulting in an infinite value for dN/dS. Six genes have dN/dS < 1 relative to the consensus sequence, but have dN/dS > 1 relative to the CC9311 genome. Whether the gene is part of the core (C) or accessory (A) genomes of *Synechococcus* is provided. The taxon containing the best tBLASTn hit with an e-value < 0.0001 is shown. For these searches, ns indicates that a significant hit was not found. Y indicates if the gene showed significant gene expression changes under one or more of the stress conditions tested in microarray experiments. N indicates that none of the stress conditions resulted in a significant change in gene expression. * indicates that the annotation of the gene was changed from “hypothetical protein” to “conserved hypothetical protein” because a significant tBLASTn hit to an ORF in another cyanobacterium was found. ^#^ indicates genes with homologues in the CC9902 genome, but the metagenome tiling to these CC9902 genes results in dN/dS < 1. ^ sync_0523 best BLASTn hits syncc9902_1875 from the CC9902 genome which has a dN/dS = 1.168 (Table 2).
