# Supplementary material for: Selection in Coastal Synechococcus (Cyanobacteria) Populations Evaluated from Environmental Metagenomes
Source: PLoS One. 2011 Sep 9;6(9):e24249. doi: 10.1371/journal.pone.0024249 (PMC3170327; doi:10.1371/journal.pone.0024249)
Supplement: Table S8 — Genes with dN/dS ratios >1 based on polymorphisms from metagenomic sequences tiled to the CC9902 genome. (DOCX) [file pone.0024249.s010.docx]

Table S8. Genes with dN/dS ratios > 1 based on polymorphisms from metagenomic sequences tiled to the CC9902 genome.

| gene | description | dN/dS | dN/dS relative to CC9902 | % of gene covered 5X /avg. read depth | core (C) or accessory (A) | best tBLASTn hit | dN/dS of CC9902 vs. BL107 |
| --- | --- | --- | --- | --- | --- | --- | --- |
| Syncc9902_1026 | hypothetical protein | 3.136 | 2.387 | 100/12.6 | A | ns | nh |
| Syncc9902_1174 | hypothetical protein | 2.933 | 2.933 | 100/22.3 | A | ns | nh |
| Syncc9902_1874 | hypothetical protein | 2.493 | 2.901 | 23/3.8 | A | ns | 0.969 |
| Syncc9902_1954 | hypothetical protein | 1.778 | 1.778 | 89/7.4 | A | ns | nh |
| Syncc9902_0240^$^ | conserved hypothetical protein* | 1.380 | 1.380 | 32/3.5 | A | *Synechococcus* CC9311 | nh |
| Syncc9902_2008 | hypothetical protein | 1.329 | 1.244 | 100/37.1 | A | ns | 1.275 |
| Syncc9902_1801 | conserved hypothetical protein* | 1.170 | 1.194 | 100/28.2 | A | *Synechococcus* CC9605 | np |
| Syncc9902_1875^ | conserved hypothetical protein* | 1.168 | 1.197 | 96/7.9 | A | *Synechococcus* CC9311 | 1.939 |
| Syncc9902_0450 | hypothetical protein | 1.099 | 1.063 | 100/60.2 | A | ns | 1.562 |
| Syncc9902_2037 | conserved hypothetical protein* | 1.005 | 1.005 | 5/1.7 | A | *Synechococcus* WH8102 | nh |
| Syncc9902_0923^#^ | conserved hypothetical protein* | Inf | Inf | 20/3.4 | C | *Synechococcus* CC9605 | 0.119 |
| Syncc9902_1576^#^ | Methylated-DNA-(protein)-cysteine S-methyltransferase | Inf | Inf | 9/3.5 | C | *Synechococcus* CC9605 | 0.108 |
| Syncc9902_2149^#^ | hypothetical protein | Inf | Inf | 8/2.0 | A | *Desulfotalea psychrophila* LSv54 | nh |
| Syncc9902_2225 | hypothetical protein | Inf | Inf | 4/1.2 | A | ns | nh |
| Syncc9902_2241^#^ | amino acid permease family protein | Inf | Inf | 15/2.0 | A | *Prochlorococcus* MIT9303 | nh |
| Syncc9902_0591 | hypothetical protein | 0.465 | 0.466 | 89/12.4 | C | *Synechococcus* CC9605 | 2.850 |
| Syncc9902_0952 | conserved hypothetical protein* | 0.216 | 0.218 | 100/26.4 | C | *Synechococcus* CC9605 | 2.075 |
| Syncc9902_1111 | Peptidase S13, D-Ala- D-Ala carboxypeptidase C | 0.150 | 0.153 | 90/13.7 | C | *Synechococcus* CC9605 | 1.038 |
| Syncc9902_1718 | hypothetical protein | 0.742 | 0.738 | 100/16.9 | A | *Synechococcus* CC9605 | 1.309 |

See Table S7 for a description of the columns. The far right column provides the dN/dS ratios between homologous genes from the CC9902 and BL107 genomes, both clade IV strains. nh indicates that there is no homologue between the two genomes. np indicates that there were no polymorphisms between the homologues. For the last 4 genes, dN/dS was < 1 from the environmental population but > 1 between CC9902 and BL107. ^#^ indicates genes with homologues in the CC9311 genome, but the metagenome tiling to these CC9311 genes results in dN/dS < 1. ^ Syncc9902_1875 best hit sync_0523 from the CC9311 genome which has a dN/dS = 2.066 (Table 1). ^$^ Syncc9902_0240 best hit sync_1115 with dN/dS = 0/0, i.e. no polymorphisms observed (Table S3).
